# Excellent survival of pathological N0 small cell lung cancer patients following surgery

**DOI:** 10.1186/s40001-023-01044-3

**Published:** 2023-02-21

**Authors:** Zichen Fu, Di Li, Chaoqiang Deng, Jingshun Zhang, Jinsong Bai, Yuan Li, Haiquan Chen, Yang Zhang

**Affiliations:** 1 Departments of Thoracic Surgery and State Key Laboratory of Genetic Engineering, Fudan University Shanghai Cancer Center, 270 Dong-An Road, Shanghai, 200032 China; 2 Institute of Thoracic Oncology, Fudan University, Shanghai, 200032 China; 3 Department of Oncology, Shanghai Medical College, Fudan University, Shanghai, 200032 China; 4 Department of Pathology, Fudan University Shanghai Cancer Center, Shanghai, 200032 China; 5 Department of Thoracic Surgery, Guanxian Xinhua Hospital, Liaocheng, 371525 China

**Keywords:** Small cell lung cancer (SCLC), Surgery, Lung cancer, Overall survival (OS), Prognosis

## Abstract

**Background:**

Current clinical guidelines recommend surgery only for cT1-2N0M0 small cell lung cancer (SCLC) patients. In light of recent studies, the role of surgery in the treatment of SCLC needs to be reconsidered.

**Methods:**

We reviewed all SCLC patients who underwent surgery from November 2006 to April 2021. Clinicopathological characteristics were retrospectively collected from medical records. Survival analysis was performed by the Kaplan–Meier method. Independent prognostic factors were evaluated by Cox proportional hazard model.

**Results:**

196 SCLC patients undergoing surgical resection were enrolled. The 5-year overall survival for the entire cohort was 49.0% (95% CI: 40.1–58.5%). PN0 patients had significantly superior survival to pN1–2 patients (*p* < 0.001). The 5-year survival rate of pN0 and pN1–2 patients were 65.5% (95% CI: 54.0–80.8%) and 35.1% (95% CI: 23.3–46.6%), respectively. Multivariate analysis revealed that smoking, older age, and advanced pathological T and N stages were independently associated with poor prognosis. Subgroup analyses demonstrated similar survival among pN0 SCLC patients regardless of pathological T stages (*p* = 0.416). Furthermore, multivariate analysis showed factors, including age, smoking history, type of surgery, and range of resection, were not independently prognostic factors for the pN0 SCLC patients.

**Conclusion:**

Pathological N0 stage SCLC patients have significantly superior survival to pN1–2 patients, regardless of features, including T stage. Thorough preoperative evaluation should be applied to estimate the status of lymph node involvement to achieve better selection of patients who might be candidate for surgery. Studies with larger cohort might help verify the benefit of surgery, especially for T3/4 patients.

**Supplementary Information:**

The online version contains supplementary material available at 10.1186/s40001-023-01044-3.

## Introduction

Small cell lung cancer accounts for 10%–15% of newly diagnosed lung cancer [1–3]. Two-thirds of the patients had extensive metastatic diseases when first diagnosed [4]. SCLC patients have a dismal prognosis due to the rapid progression, with a 5-year survival rate of less than 7% [5]. Given the strong association with smoking [4, 6, 7], the incidence of SCLC is unlikely to decline in East Asia in the next few years because of the high smoking rates [8]. Considering the high incidence and poor prognosis of SCLC, attention to its management is required in order to improve the survival of the patients.

A high proportion of SCLC patients do not meet the indication of surgical treatment when diagnosed [9], surgery being the recommended option for cT1-2N0M0 tumors [1], which benefit most from surgical resection [12–14, 20]. Chemotherapy with concurrent radiotherapy is the recommended treatment by clinical guidelines for these patients. Yet recent non-randomized data indicate better prognosis of patients with more advanced diseases receiving surgical resection, suggesting that the role of surgery in the treatment of SCLC needs to be reconsidered [13, 14, 21].

Thus, in this study, SCLC patients who underwent resection were reviewed with a view to reevaluate the role of surgery in SCLC treatment. Postoperative survival and prognostic factors were investigated, including the benefit of postoperative adjuvant therapy.

## Methods

### Patients

We reviewed continuous patients who underwent radical surgery from November 2006 to April 2021 at the Department of Thoracic Surgery, Fudan University Shanghai Cancer Center (FUSCC), Shanghai, China. Patients who (1) had a preoperative diagnosis of lung cancer, (2) met the indications for surgery, (3) and with histologically confirmed SCLC after surgical resection were collected. After the routine examination, including chest CT, abdomen ultrasound, and cranial MRI, T1-2N0M0 patients diagnosed with SCLC by preoperative puncture biopsy might be recommended for surgical resection. And for patients without the diagnosis of puncture biopsy, surgery might be considered as long as the disease was in limited stage. In total, 196 SCLC patients were enrolled. Clinicopathologic data, including sex, age, smoking history, radiological findings, time of surgery, surgery procedures, pathology reports, and postoperative adjuvant therapy regimens, were collected from medical records. Postoperative adjuvant treatment modalities were classified as non-adjuvant therapy, adjuvant chemotherapy, and adjuvant chemoradiotherapy. The adjuvant chemoradiotherapy was defined as receiving chemotherapy followed by radiotherapy after surgery, and radiotherapy, including chest radiation, prophylactic cranial irradiation, or both. All pathologic sections were re-reviewed by 2 pathologists. The clinical and pathological staging was reevaluated according to the American Joint Committee on Cancer (AJCC) eighth edition TNM and the Veterans Administration Lung Study Group (VALSG) staging systems.

For preoperative staging, all patients underwent computer tomography (CT) scans of the chest and the abdomen, ultrasonography of abdominal and cervical/ supraclavicular regions, magnetic resonance imaging, or CT scans of the brain and single-photon emission computerized tomography (SPECT) of the bone. Since health insurance in China did not cover positron emission tomography with fluorodeoxyglucose (FDG-PET), FDG-PET was optional. Endobronchial ultrasound-guided transbronchial needle aspiration (EBUS-TBNA) or mediastinoscopy was applied for suspected mediastinal lymph node involvement whenever possible.

This research was conducted in accordance with the amended Declaration of Helsinki and was approved by the Ethics Committee and Institutional Review Boards of FUSCC (IRB2008223-9, Date: 2020/07/14), and all patients were exempt from an informed consent due to the retrospective nature of this study.

### Statistical analysis

Data are presented as means, medians, or counts and percentages, as appropriate. The OS was defined as the time from surgery to death from any cause or last follow-up. Kaplan–Meier method was used to estimate OS, with a log-rank test to evaluate significant differences. Independent prognostic factors were evaluated by Cox proportional hazard model. All factors with a *p* < 0.05 in the univariate analysis were enrolled in the multivariate analysis. All of the tests were conducted bilaterally, and statistical significance was *p* < 0.05. Both simple percentage and a weighted Cohen’s kappa coefficient were calculated in order to assess the accuracy of clinical staging. Power calculation was performed with PASS 2021. All statistical analysis was performed by IBM SPSS 25.0(IBM-SPS Inc., Armonk, NY) and GraphPad Prism 9.

## Results

### Patient characteristics

In total, 196 SCLC patients who underwent surgical resection were reviewed (Table 1), consisting of 167 male patients (85.2%) and 29 female patients (14.8%), with a median age of 63.0 (Range: 36.0–84.0) years. 149 (76.0%) of the patients had a history of smoking. 149 of the patients (76.0%) underwent the surgery by thoracotomy. And 144 of the patients (73.5%) underwent lobectomy. Of all patients, 82 (41.8%), 45 (23.0%), and 69 (35.2%) patients were diagnosed with pathological stages I, II, and III, respectively. The postoperative pathological evaluation indicated 101 (50.5%) patients with lymph node metastases. 37 (18.9%) patients were preoperatively diagnosis with SCLC by biopsy. 80 (40.8%) patients received adjuvant chemotherapy and 65 (33.2%) patients received adjuvant chemoradiotherapy as postoperative treatment, whereas 51 (26.0%) patients did not undergo any postoperative adjuvant therapy because of preference or the intolerance of side effects. The median follow-up time was 3.2 years (95%CI: 2.2–4.1), and the median OS was 4.6 years (95%CI: 2.3–7.0). Power of the cohort was 0.804.

**Table 1:** Characteristics of the patients

Characteristics	*N* = 196(%)
Age, median(range)	63(36–84)
Gender, N(%)	
Male	167(85.2)
Female	29(14.7)
Respiratory risk, n (%)	
Active smoker	149(76.0)
Never smoker	47(23.9)
Cancer description	
Median size cm (min–max)	2.8(0.7–11.2)
Surgery, n(%)	
Video-assisted thoracoscope surgery	47(23.9)
Thoracotomy	149(76.0)
Range of resection, n (%)	
PNEUM	10(5.1)
SLE	3(1.5)
BILOB	20(10.2)
LOB	144(73.4)
SEG	10(5.1)
WED	9(4.5)
pT staging, n (%)	
pT1	105(53.6)
pT2	67(34.2)
pT3	19(9.7)
pT4	5(2.6)
pN staging, n (%)	
pN0	95(48.5)
pN1	36(18.4)
pN2	65(33.2)
pTNM staging, n (%)	
IA	65(33.2)
IB	17(8.7)
IIA	6(3.1)
IIB	39(19.9)
IIIA	54(27.6)
IIIB	15(7.6)
cT staging, n (%)	
pT1	104(53.1)
pT2	53(27.0)
pT3	14(7.1)
pT4	6(3.1)
Unknown	19(9.7)
cN staging, n (%)	
pN0	86(43.9)
pN1	37(18.9)
pN2	54(27.6)
Unknown	19(9.7)
cTNM staging, n (%)	
IA	52(26.5)
IB	19(9.7)
IIA	6(3.1)
IIB	39(19.9)
IIIA	56(28.6)
IIIB	5(2.6)
Unknown	19(9.7)
Postoperative therapy	
Non-adjuvant therapy	58(29.6)
Adjuvant chemotherapy	75(38.3)
Adjuvant chemoradiotherapy	63(32.1)
Time from diagnosis to surgery (days)	30(5–719)
BMI(kg/m^2^)	24.2(17.0–35.3)
ECOG score	
1	16
0	180

Values are presented as n (%)

Unknown: Data was not available

*PNEUM* pneumonectomy, *SLE* Sleeve resection, *BILOB* Bilateral lobectomy, *LOB* Lobectomy, *SEG* Segmentectomy, *WED* Wedge resection, *BMI* Body mass index, *ECOG* Eastern Cooperative Oncology Group

### Survival analyses for surgically resected SCLC patients

**Fig. 1:**
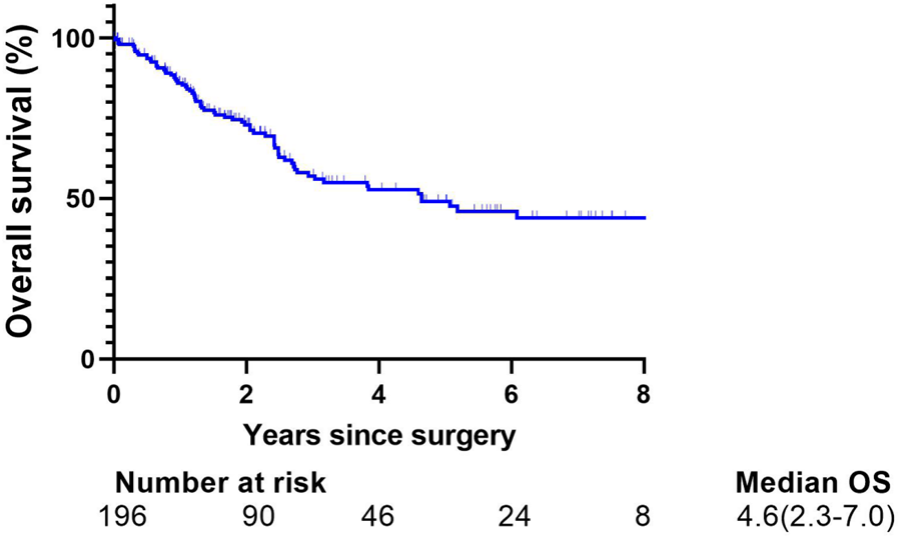
Overall survival after resection for all 196 patients with SCLC

Survival analysis showed that the 5-year OS rate of the entire cohort was 49.0% (95%CI: 40.1%–58.5%) (Fig. 1).

Univariate analyses found that female patients and non-smokers had a superior prognosis (*p* = 0.003 and *p* = 0.005, power: 0.998) (Additional file 1: Fig. S1a and B).

**Fig. 2:**
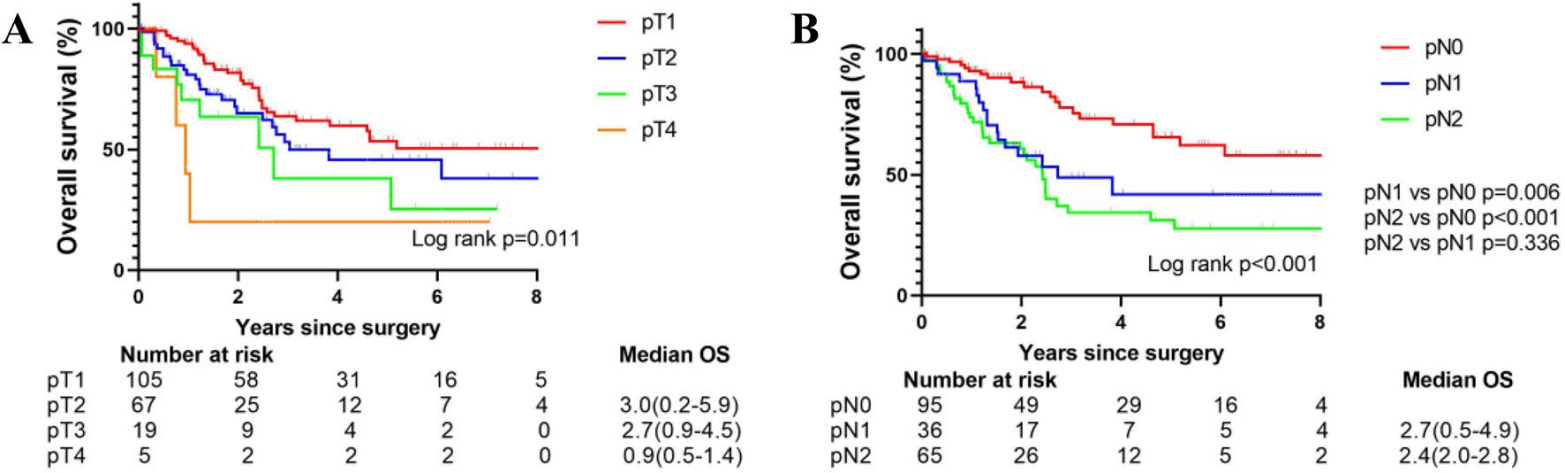
Overall survival after resection for all patients stratified by pT (2A) and pN (2B) stages

Statistical differences were observed between the prognosis of patients with different pathological T stages (*p* = 0.011, power: 0.973) (Fig. 2A).

The survival of patients with different pathological N stages was also statistically different (*p* < 0.001, power: 0.973) (Fig. 2B). However, Kaplan–Meier survival curves of pathological N1 and N2 patients were similar (*p* = 0.336, power: 1.000). The 2-year survival rate of pN0, pN1, and pN2 patients was 88.3% (95% CI: 82.8–98.1%), 57.8% (95% CI: 43.0–76.4%), and 60.8% (95% CI: 49.0–75.5%), respectively. And the 5-year survival rate of pN0–2 patients are 65.5% (95% CI: 54.0–80.8%), 41.9% (95% CI: 23.1–61.7%), and 31.2% (95% CI: 16.7–44.3%), respectively. For the entire cohort, multivariate Cox regression indicated that lymph node metastasis was related to poor prognosis (HR = 3.171, 95%CI: 1.892–5.314, *p* < 0.001). Meanwhile, pT2-4 stage (HR = 1.687, 95%CI: 1.043–2.730, *p* = 0.033), positive smoking history (HR = 2.305, 95%CI: 1.110–4.785, *p* = 0.025), and older age (HR = 1.034, 95%CI: 1.003–1.066, *p* = 0.031) were also related to worse survival (Table 2).

**Table 2:** Univariate and multivariate analyses of factors associated with overall survival for surgically resected SCLC patients

Parameters	Univariate analysis		Multivariate analysis	
	Hazard ratio(95%Cl)	P value	Hazard ratio(95%Cl)	P value
N1–2 vs N0	2.851(1.709–4.756)	** < 0.001**	3.114(1.858–5.219)	** < 0.001**
T2–4 vs T1	1.745(1.093–2.784)	**0.020**	1.662(1.028–2.688)	**0.038**
Smoking history	2.610(1.293–5.271)	**0.007**	2.305(1.110–4.785)	**0.025**
Open vs VATS	1.075(0.588–1.968)	0.814		
Extent of resection				
LOB vs < LOB	1.665(0.606–4.576)	0.323		
> LOB vs < LOB	0.766(0.171–3.434)	0.728		
Age	1.032(1.002–1.064)	**0.037**	1.035(1.003–1.068)	**0.033**
Time from diagnosis to surgery	1.520(0.916–2.522)	0.105		
ECOG score	1.665(0.761–3.645)	0.202		
BMI	0.992(0.558–1.763)	0.978		

Only parameters with statistical significance in univariate analysis were displayed in multivariate analysis

*VATS* Video-assisted thoracic surgery, *LOB* lobectomy, *BMI* Body mass index, *ECOG* Eastern Cooperative Oncology Group

*p* < 0.05 is indicated by bold

### Subgroup analyses of pN0 and pN1–2 patients

**Fig. 3:**
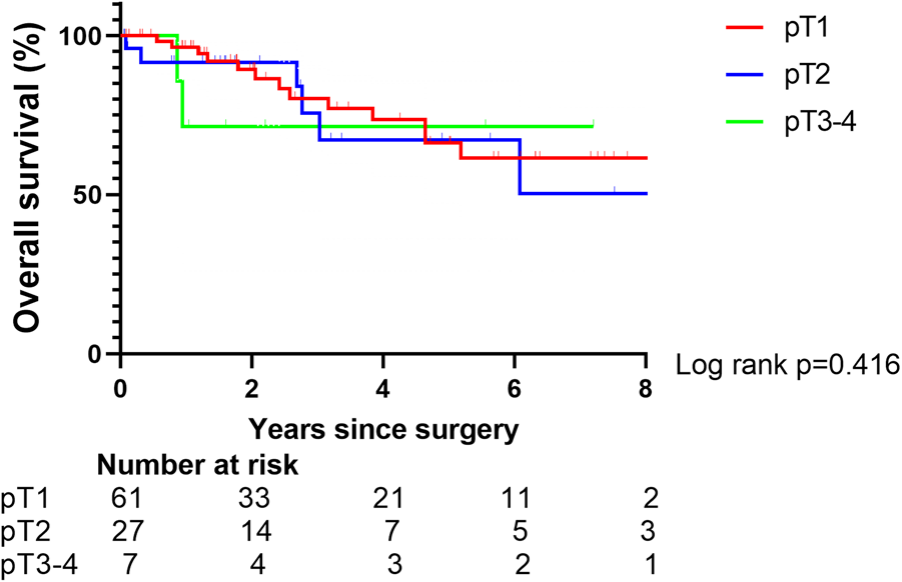
Overall survival after resection for pN0 patients stratified with pT stage

Among the patients without lymph node metastasis, no statistical difference in survival was observed among patients with different pathological T stages (*p* = 0.416, power: 0.275) (Fig. 3).

Similar survival was also observed among patients stratified by tumor size (*p* = 0.686, power: 0.274) (Additional file 2: Fig. S2).

**Fig. 4:**
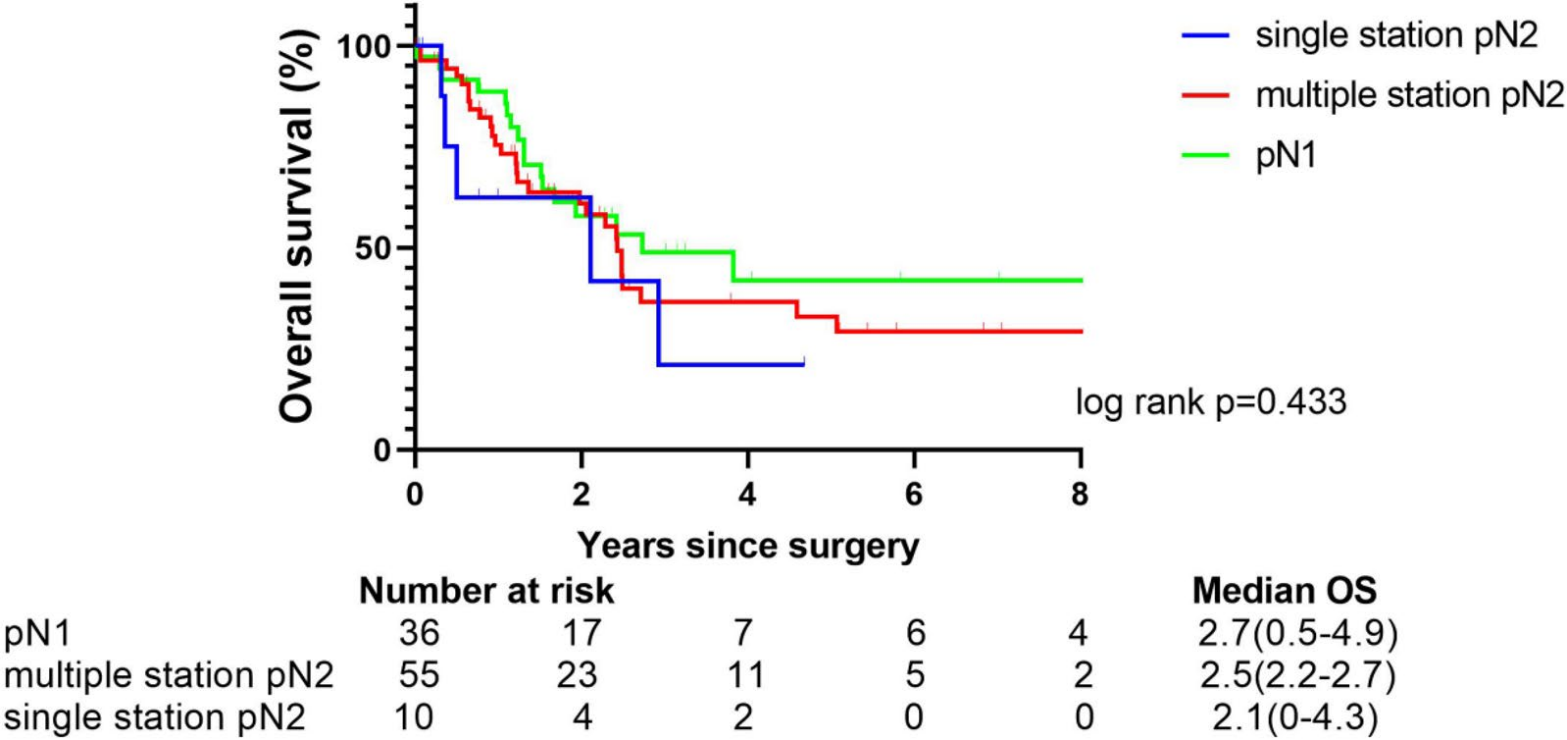
Overall survival after resection for pN1–2 patients with different lymph node metastasis statuses

Furthermore, no independent prognostic factor was revealed by univariate cox regression analysis for pN0 patients (Table 3). For the patients with lymph node metastasis, the prognosis of patients diagnosed with pN1, single-station pN2, and multiple-station pN2 were similar (*p* = 0.433, power: 0.392) (Fig. 4).

**Table 3:** Univariate analysis of factors associated with overall survival for surgically resected SCLC patients among pN0 patients

Parameters	Univariate analysis	
	Hazard ratio(95%Cl)	*p* value
T2–4 vs T1	1.249(0.517–3.016)	0.622
Smoking history	35.056(0.521–2358.193)	0.098
Open vs VATS	1.695(0.495–5.800)	0.401
Extent of resection		
> LOB vs < LOB	0.860(0.201–3.822)	0.876
LOB vs < LOB	0.729(0.101–5.250)	0.754
Age	1.051(0.980–1.126)	0.164
Time from diagnosis to surgery	1.422(0.550–3.676)	0.467
ECOG score	1.375(0.178–10.596)	0.760
BMI	1.649(0.519–5.236)	0.396

*VATS* Video-assisted thoracic surgery, *LOB* lobectomy, *BMI* Body mass index, *ECOG* Eastern Cooperative Oncology Group

### Impact of adjuvant therapy on survival for surgically resected SCLC patients with stage pN0

**Fig. 5:**
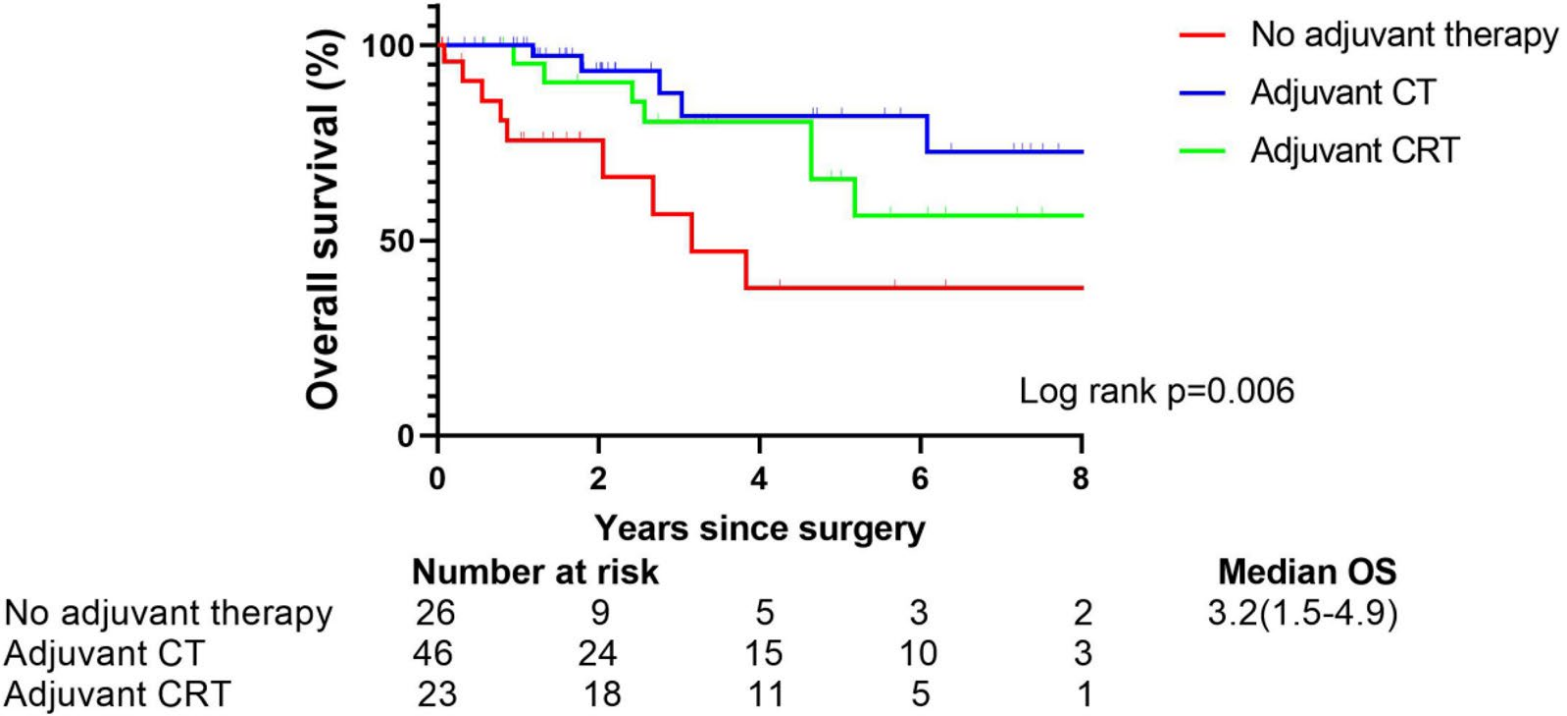
Overall survival after resection for N0 patients stratified by receipt of adjuvant therapy

Patients with postoperative adjuvant therapy had a superior prognosis than patients without any adjuvant treatment (*p* = 0.006). The Kaplan–Meier survival curves of the patients receiving adjuvant chemotherapy and adjuvant chemoradiotherapy were similar (*p* = 0.271, power: 1.000) (Fig. 5).

## Discussion

Clinical practice guidelines recommend that surgical resection as surgical treatment be considered only for stage I–IIA SCLC patients (T1-2N0M0) [1]. Yet variation exists in the use of surgical therapy for early SCLC [22]. We have noticed that some patients diagnosed with SCLC had a better prognosis than expected after surgical resection. Therefore, to reevaluate the role of surgery in SCLC treatment, we retrospectively reviewed SCLC patients undergoing surgical resection. After analysis, we found that pN0 patients had significant superior survival than pN1–2 patients, regardless of other features. Furthermore, receiving postoperative adjuvant therapy was associated with better prognosis among pN0 SCLC patients who underwent surgical resection.

Previous studies have emphasized the benefits of surgery as a treatment modality for SCLC patients [9, 15]. Chen et al. found that pathological stage I–IIA SCLC patients who underwent surgery showed a better prognosis than those who did not (HR = 0.292, 95%CI:0.237–0.361, *p* < 0.001) [15]. Uprety and colleagues indicated that clinical T1-2N0M0 SCLC patients who underwent surgery and chemotherapy showed a superior survival than patients with chemoradiation, and the median OS of both groups was 61.7 months (95%CI: 51.8–76.5) and 31.2 months (95% CI: 26.3–37.0), respectively [9]. In the study of Chai, 5576 clinical T1-4N2M0 SCLC patients were investigated based on the SEER database, finding that the median OS of patients who underwent surgery was significantly longer than the non-surgical patients (20 vs. 15 months, *p* = 0.0024) and that patients with a staging of T1N2 who underwent surgical resection showed better prognosis (HR = 0.565, 95%CI: 0.401–0.798, *p* = 0.001) [19]. Wakeam and colleagues retrospectively reviewed the prognosis of 29,994 SCLC patients, indicating that surgical resection is associated with significantly longer survival for early SCLC [23]^.^ These studies were limit to specific stages of the disease. We found that among the patients who underwent surgical resection, the prognosis of pN0 patients was significantly superior to pN1–2 patients (*p* < 0.001). The 5-year survival rate of pN0 patients and pN1–2 is 65.5% (95% CI: 54.0–80.8%) and 35.1% (95% CI: 23.3–46.6%), respectively. Moreover, we discovered smoking, older age, and more advanced pathological T stage were also independent factors associated with poor prognosis.

Several previous studies suggested it was SCLC patients with early T and N stage who benefit more from surgical resection [16, 17]. Wei and colleagues investigated the benefits of surgery. Finding that the 5-year OS rate of patients underwent surgery was better compared to non-surgical patients (36.7% vs. 16.8%, *p* < 0.001). Yet the improvement of prognosis was less significant for T3 patients or patients with a tumor over 5.0 cm [17]. Gu and colleagues compared the prognosis between patients, who received comprehensive treatment, including surgery and chemoradiotherapy, and patients treated with only chemoradiotherapy. The results revealed that only patients with pathological stage I and II SCLC among the comprehensive treatment group showed significantly better survival than the chemoradiotherapy group [16]. We stratified the patients by gender, smoking history, pathological T staging, and status of lymph node metastasis, revealing that statistical differences were found between the patients divided by pathological T and N stages, gender, and different smoking history. Among the pN0 patients, the Kaplan–Meier survival curves of patients classified by different pathological T stages (*p* = 0.416) or tumor sizes (*p* = 0.686) both resulted in similar trends. Multivariate Cox regression of pN0 patients indicated no other independent factor related to prognosis, including pathological T stage. This suggests that the survival of pN0 SCLC patients might be similar and that other variables, including pT staging, is less associated with the prognosis among the N0 patients. The OS of pN1–2 patients indicated no statistical difference between the prognosis of pN1, single-station pN2, and multiple-station patients (*p* = 0.433). We supposed that pN0 patients might have a superior prognosis than pN1–2 patients. However, pN0 patients might have similar prognosis, regardless of features, such as pathological T stage. All patients without lymph node metastasis, including pT3 and pT4 patients, showed similar excellent survivals after surgery. These results indicate that occult systemic spread is the most important factor governing the poor prognosis of SCLC patients. Hence, the status of lymph node metastases matters more to the survival because it reflects the status of occult systemic spread of the tumor. Hence, the pN stage is the most important factor for predicting the extent to which patients will benefit from surgery. Overall, findings of this study suggest that all pN0 patients regardless of T stage should be reconsidered for surgical resection.

The discrepancy between clinical and pathological staging has been observed in multiple previous studies [18, 19]. Ahn et al. revealed pooled proportions of agreement between clinical and pathological T stage of 42.1% (95% CI, 37.7–46.6%) to 50.0% (95% CI, 45.5–54.5%) [18]. Hart and colleagues investigated the discrepancy between clinical and pathological staging of lung adenocarcinoma, indicating that 57.5% cases showed pathological T stage differed from clinical T stage [19]. Considering the pN0 patients showed superior prognosis to the pN1–2 patients, we assumed that patients without lymph node metastases might benefit from surgery. Therefore, we propose that more thorough preoperative evaluation and precise clinical staging of SCLC patients be performed to identify patients with potential lymph node metastases, so that cN0 patients be offered potentially beneficial surgery, leading to a better prognosis.

There are several limitations of our study. Selection bias, information bias and institutional bias could not be avoided as this is a single-center study that was conducted retrospectively. The number of pT3/4 patients were less comparing to that of pT1/2 patients. There were only 1 patient diagnosed with pT4 stage and 6 patients with pT3 disease among the pN0 group reviewed, especially. Patients with less advanced N stage were mostly diagnosed with less extent of invasion. And the T3/4 patients of the cohort actually received the resection accidentally. The patients had a preoperative diagnosis of non-small cell lung cancer with SCLC revealed by postoperative pathology. Adequately powered statistical comparisons might be difficult to make because of the low numbers of patients of certain strata. In order to reduce the impact on results due to randomness, the pT4 and pT3 patients were combined into same group when analyzing the pN0 patients stratified with different T staging. Studies with large cohort might help verify the conclusion, especially for patients with advanced T staging. Besides, the tests of survival of N0 patients with different T staging showed low power. The cohort contained few T3/4 patients, especially the T4N0 patients, which might be relevant with the low power. And the less significant difference of the survival of N0 patients stratified by T staging might also lead to lower power of the test. Studies with larger cohort, especially T3/4 patients might help reduce the bias, improve the power of test, and verify the related conclusion.

## Conclusion

In conclusion, investigation of the survival of SCLC patients following surgical resection indicated SCLC patients without pathological lymph node metastases has significantly superior survival following surgical resection compared to patients with lymph node metastases, regardless of other features, including pathological T stage. And pN0 patients showed favorable prognosis. Thorough preoperative evaluation, such as PET-CT and lymph node FNAC/biopsy, should be performed to estimate the status of lymph node metastases before starting treatment. Patients with no evidence of lymph node involvement could be considered for surgery. Yet there were few pT3/4 patients reviewed in this study, so that the benefit of surgical resection for T3/4N0 patients needs to be validated. More prospective studies are required to validate these results. Studies with larger cohort, especially T3/4 patients might help verify the benefit of surgery, especially for advanced T staging.

## Supplementary Information

**Additional file 1: Fig. S1.** Overall survival after resection for patients stratified by gender (1A) and smoking history (1B).

**Additional file 2: Fig. S2.** Overall survival after resection for pN0 patients stratified by tumor size.

## Data Availability

The authors confirm that the data supporting the findings of this study are available within the article and its supplementary materials.

## Abbreviations

AJCC: American Joint Committee on Cancer
CT: Computer tomography
EBUS-TBNA: Endobronchial ultrasound-guided transbronchial needle aspiration
FDG-PET: Fluorodeoxyglucose-positron emission tomography
OS: Overall survival
SCLC: Small cell lung cancer
VALSG: The Veterans Administration Lung Study Group
FNAC: Fine needle aspiration cytology
SPECT: Single-photon emission computerized tomography
