# Supplementary figures and images for: Excellent survival of pathological N0 small cell lung cancer patients following surgery
Source: Eur J Med Res. 2023 Feb 21;28:91. doi: 10.1186/s40001-023-01044-3 (PMC9942372; doi:10.1186/s40001-023-01044-3)

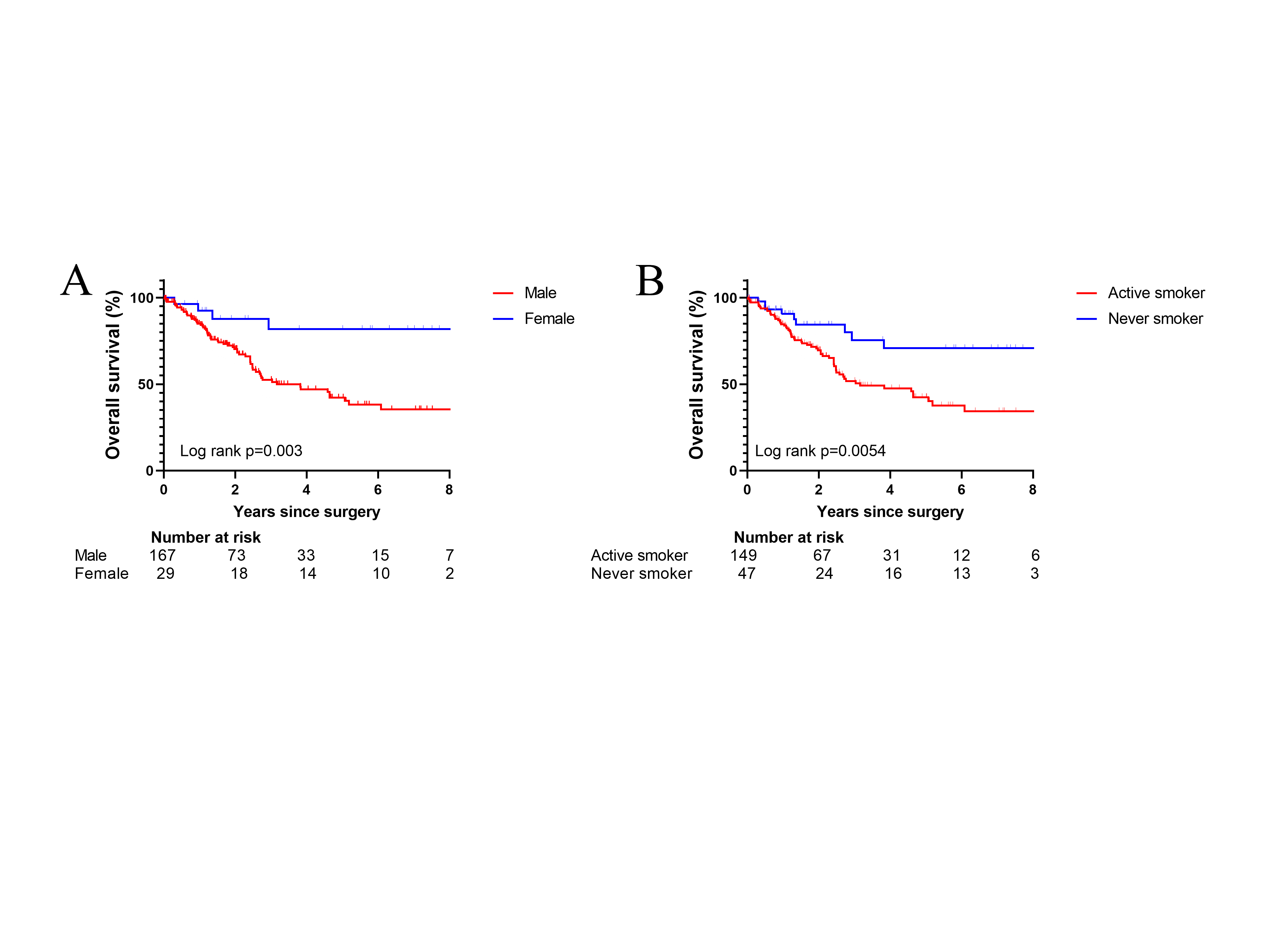

Supplement: Supplementary file 1 — Additional file 1: Fig. S1. Overall survival after resection for patients stratified by gender (1A) and smoking history (1B). [file 40001_2023_1044_MOESM1_ESM.tif]

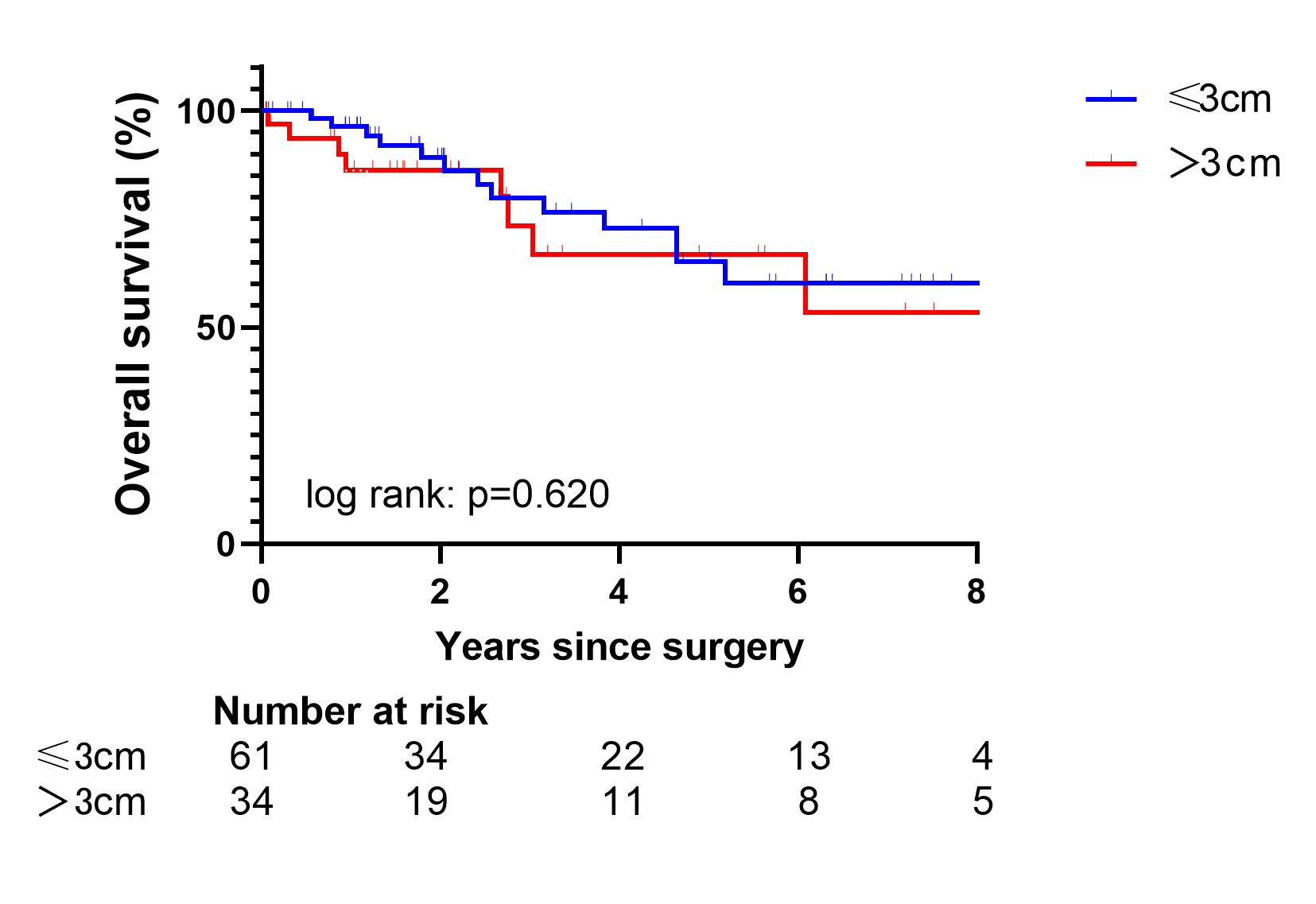

Supplement: Supplementary file 2 — Additional file 2: Fig. S2. Overall survival after resection for pN0 patients stratified by tumor size. [file 40001_2023_1044_MOESM2_ESM.tif]
